# Supplementary material for: From bud formation to flowering: transcriptomic state defines the cherry developmental phases of sweet cherry bud dormancy
Source: BMC Genomics. 2019 Dec 12;20:974. doi: 10.1186/s12864-019-6348-z (PMC6909552; doi:10.1186/s12864-019-6348-z)
Supplement: Supplementary file 3 — Additional file 3. Additional information on the model optimization. [file 12864_2019_6348_MOESM3_ESM.pdf]

Additional File 3. Additional information on the model optimization

Multinomial logistic regression (LR)

The LR models were optimized using the *LogisticRegressionCV* function with default parameters, multi_class: ‘multinomial’, max_iter: 1000 and the ‘lbfgs’ solver for the optimization.

```
LogisticRegressionCV(cv =5 , multi_class='multinomial', solver='lbfgs', max_iter=1000)
```

Multi-layer perceptron – MLP

The MLP models were optimized using the *MLPClassifier* function and the *GridSearchCV* function for cross-validation.

```
param_grid = {'alpha':[1e-3, 5e-3, 1e-4, 5e-4], 'learning_rate_init': [0.005, 0.01, 0.1]}
GridSearchCV(MLPClassifier(hidden_layer_sizes=(24,8),
                           max_iter = 1000,
                           solver = 'adam',
                           verbose = 0,
                           tol = 1e-8,
                           random_state = 1),
             param_grid, cv=5, n_jobs = -1)
```

K-nearest neighbor classifier – KNN

The KNN models were optimized using the *neighbors.KNeighborsClassifier* function and the *GridSearchCV* function for cross-validation.

```
param_grid = {'n_neighbors' : [3,5,11,19],
              'weights' : ['uniform', 'distance'],
              'metric' : ['euclidean', 'manhattan']}
GridSearchCV(neighbors.KNeighborsClassifier(kernel = ‘rbf’),
             param_grid, cv=5, n_jobs = -1)
```

Support vector machine – SVM

The SVM models were optimized using the *SVC* function and the *GridSearchCV* function for cross-validation.

```
param_grid = {'C':[5e2, 1e3, 5e3, 1e4, 5e4, 1e5],
              'gamma':[0.0001, 0.0005, 0.001, 0.005, 0.01, 0.1]}
GridSearchCV(SVC(kernel = ‘linear’),
             param_grid, cv=5, n_jobs = -1)
```

Random forest– RF

The RF models were optimized using the *RandomForestClassifier* function and the *GridSearchCV* function for cross-validation.

```
param_grid = {'n_estimators': [320,330,340],
              'max_depth': [8, 9, 10, 11, 12],
              'random_state': [0]}
GridSearchCV(RandomForestClassifier( ),
             param_grid, cv=5, n_jobs = -1)
```
